# The use of capnography for real-time monitoring of mask ventilation during induction of general anaesthesia: A prospective observational study

**DOI:** 10.1097/EA9.0000000000000009

**Published:** 2022-11-04

**Authors:** Taiichiro Hayashida, Natsuko Nozaki-Taguchi, Shin Sato, Takumi Meguro, Yasunori Sato, Shiroh Isono

**Affiliations:** From the Department of Anaesthesia, Pain and Palliative Care Medicine, Chiba University Hospital (TH, SS, TM), Department of Anesthesiology, Graduate School of Medicine, Chiba University, Chiba (NNT, SI) and Department of Preventive Medicine and Public Health, Keio School of Medicine, Tokyo, Japan (YS)

Editor,

Effective mask ventilation with proper airway manoeuvres secures maintenance of pulmonary ventilation and oxygenation during induction of general anaesthesia.^1^ However, life-threatening difficulties and impossible mask ventilation do occur. The difficult airway guidelines of the American Society of Anesthesiologists and the Difficult Airway Society do not propose any objective criteria for diagnosis of difficult and impossible mask ventilation.^2,3^ Therefore, real-time breath-by-breath monitoring of mask ventilation effectiveness could maximise the risk management strategies of difficult airway guidelines.^2–5^

Capnography monitoring is routinely used during anaesthesia induction for confirmation of tracheal intubation. The rising trace of the capnogram (phase II) reflects the change in the expired gas composition because of mixing of the dead space (anatomical and physiological) gas and alveolar CO_2_. Furthermore, the end-tidal CO_2_ concentration (ET CO_2_) during phase II theoretically changes from zero to alveolar CO_2_ concentration, which may possibly reflect the tidal volume.^6^

The aim of this prospective observational study was to test the hypothesis that tidal volume during mask ventilation can be estimated by the combination of the capnogram waveform and ET CO_2,_ enabling the determination of threshold ET CO_2_ values, which indicate critical and secure ventilation, defined as a tidal volume less than 2 ml kg^−1^ and greater than 5 ml kg^−1^ ideal body weight (IBW).

Following ethical approval (Ethical Committee of Graduate School of Medicine, Chiba University, Chiba, Japan; number 3203) and clinical study registration (UMIN000035108, 3 December 2018: https://upload.umin.ac.jp/cgi-open-bin/ctr_e/ctr_view.cgi?recptno=R000039815), written informed consent was obtained from each patient. Between 3 December 2018 and 21 January 2020, adult patients scheduled for surgery under general anaesthesia were screened. The following patients were excluded: candidates for awake intubation, full stomach, undergoing emergency surgery, tracheostomy and tracheoesophageal fistula, dementia and with allergy for rocuronium or propofol.

After loss of consciousness, rocuronium 0.7 mg kg^−1^ was injected. The anaesthetist performed one handed mask ventilation through an anaesthesia full-face mask for 1 min as usual but was instructed not to exceed a peak airway pressure of 20 cmH_2_O. Twelve anaesthesia providers with a wide range of experience participated in this study without any prior knowledge of the purpose of the study.

Expiratory tidal volume (GF-220R Multigas/Flow unit, Nihon Kohden, Tokyo, Japan), and ET CO_2_ concentration with a mainstream capnometer (CAP-ONE, TG-970P, Nihon Kohden, Tokyo, Japan, accuracy: ±2 mmHg) were continuously measured. Three distinct capnography waveforms were identified: plateaued capnogram, upswing capnogram (lack of the phase III plateau) and flat-line capnogram (ET CO_2_ zero). The monitoring screen and video images of the patient's head, face and neck, and the operating room were integrated synchronously on the same screen, and these were recorded to a hard disk for later detailed breath-by-breath analyses of the ventilation variables.

On the basis of our previous study,^7^ the sample size was determined to be 52 breaths for each upswing capnogram category assuming *α* = 0.05 (two tailed), *β* = 0.8 to detect a clinically meaningful ET CO_2_ difference of 4 mmHg. We theorised that 50 patients could produce more than 52 breathes for each capnogram category during anaesthesia induction.

Two-thirds of all upswing capnogram breaths were randomly selected, and receiver operating characteristic (ROC) curves and optimal cutoff values were estimated. The remaining one-third of traces were used as a validation dataset for the cutoff value, and we calculated sensitivity, specificity and AUC. A value of *P* less than 0.05 was considered as statistically significant, and all *P* values were two-sided. All statistical analyses were performed using SAS Ver. 9.4 (SAS Institute, Cary, North Carolina, USA).

From a total of 1219 breaths in 50 participants (predominantly nonobese females), capnography waveform classification was possible in 1215 breaths: these included 920 (76%) plateaued, 244 (20%) upswing and 51 (4%) flat-line capnograms. A plateaued capnogram had moderate diagnostic performance of secure ventilation with 85% sensitivity and 81% specificity. A flat-line capnogram had a highly specific diagnostic performance of critical hypoventilation with 60% sensitivity and 99% specificity. In contrast, an upswing capnogram alone had a poor diagnostic performance for both secure and critical ventilation.

**Fig. 1:**
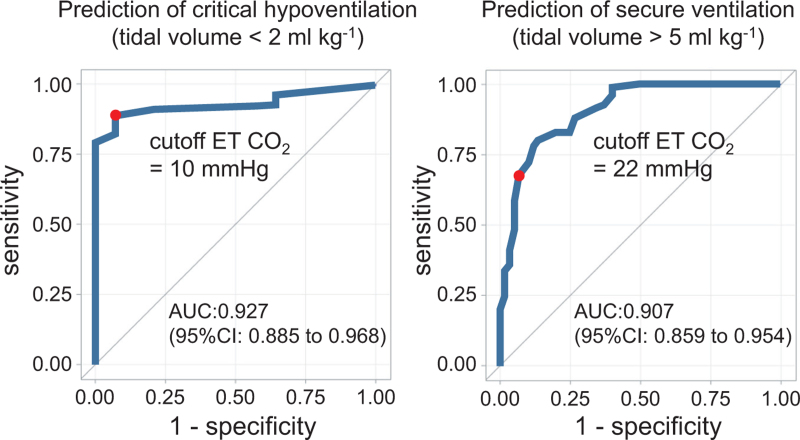
Results of receiver operating curve analyses of upswing capnography waveform breaths (*n* = 244) for prediction of adequacy of ventilation.

From the 244 upswing capnogram breaths, 164 breaths and 80 breaths were randomly selected for ROC analyses and cross validation tests. Figure 1 presents the ROC analysis result for prediction of critical and secure ventilation in upswing capnogram breaths and determines the cutoff ET CO_2_ values to be 10 and 22 mmHg with AUC values of 0.927 and 0.907, respectively. Using the cutoff values, cross validation test resulted in 82% sensitivity and 67% specificity for critical hypoventilation prediction, and 67% sensitivity and 93% specificity for secure ventilation prediction.

Left, critical hypoventilation (defined as a tidal volume less than 2 ml kg^−1^ ideal body weight); right, secure ventilation (defined as a tidal volume greater than 5 ml kg^−1^ ideal body weight). AUC, area under the receiver operating curve; CI, confidence interval; ET CO_2_, end tidal carbon dioxide concentration in expired breath.

Although tidal volume can be measured directly by various monitors, a threshold for critical hypoventilation volume should be individualised by the patient's body size: the difficulty in such tidal volume normalisation may limit the clinical usefulness of monitoring absolute tidal volume for on-site decision making. This study is the first to systematically test the performance of the capnogram and ET CO_2_ for monitoring the effectiveness of mask ventilation. Unlike tidal volume, the normal ET CO_2_ range, while depending on cardiac output and body temperature, is independent of age and body size.

On the basis of the findings of this study, we propose a simple decision-making algorithm by using the combination of the capnogram and ET CO_2_ during induction of general anaesthesia. First, either a plateaued capnogram or an upswing capnogram with ET CO_2_ greater than 22 mmHg guarantees secure mask ventilation with oxygenation. When the ET CO_2_ in an upswing capnogram is between 10 and 22 mmHg, the anaesthesiologist should try to maintain ET CO_2_ at above 22 mmHg by using advanced airway techniques such as insertion of an oral airway, and/or two-handed mask ventilation. If ET CO_2_ cannot be increased by these interventions, the anaesthesiologist needs to consider the use of the difficult airway algorithm, including supraglottic airway insertion, wakening the patient and surgical cricothyroidotomy. When there is no capnogram waveform or ET CO_2_ less than 10 mmHg in an upswing capnogram is persistent, then the difficult airway algorithm should be commenced immediately.

In conclusion, the combination of a capnogram and ET CO_2_ concentration accurately estimated a normalised tidal volume. ET CO_2_ greater than 22 mmHg is the minimum target for achieving well tolerated and effective mask ventilation during induction of general anaesthesia. The clinical usefulness of the proposed real-time mask ventilation grading system needs to be tested in future large-scale multicentre studies, including patients with difficult airway.
